# Study protocol of a double-blind randomized control trial of transcranial direct current stimulation in post-stroke fatigue

**DOI:** 10.3389/fneur.2023.1297429

**Published:** 2024-01-29

**Authors:** Wai Kwong Tang, Hanna Lu, Thomas Wai Hong Leung, Jong S. Kim, Kenneth Nai Kuen Fong

**Affiliations:** ^1^Department of Psychiatry, Chinese University of Hong Kong, Shatin, Hong Kong SAR, China; ^2^Department of Medicine and Therapeutics, Chinese University of Hong Kong, Shatin, Hong Kong SAR, China; ^3^Department of Neurology, Kangneung Asan Hospital, University of Ulsan, Ulsan, Republic of Korea; ^4^Department of Rehabilitation Sciences, Hong Kong Polytechnic University, Hung Hom, Hong Kong SAR, China

**Keywords:** stroke, transcranial direct current stimulation (tDCS), rehabilitation, post-stroke fatigue (PSF), randomized control trial (RCT)

## Abstract

**Rationale:**

Post-stroke fatigue (PSF) is a frequent problem in stroke survivors and often hinders their rehabilitation. PSF is difficult to treat, and pharmacological therapy is often ineffective. Transcranial direct current stimulation (tDCS) can modulate motor, sensory, cognitive and behavioral responses, as it alters neuronal activity by delivering a small amount of current via the scalp to the cortex, resulting in prolonged alterations to brain function. tDCS has been studied for the treatment of fatigue associated with other neurological diseases, namely, multiple sclerosis, Parkinson’s disease and post-polio syndrome.

**Aims:**

This proposed project will examine the effect of tDCS on PSF.

**Sample size estimates:**

We will recruit 156 participants aged 18 to 80 with chronic stroke and allocate them equally to two groups (i.e., *n* = 78 per group).

**Methods and design:**

This proposed project will be a double-blind randomized control trial. The participants will be randomly divided into two groups. The control group will receive sham tDCS, and the treatment group will receive active tDCS. The latter treatment will involve application of a constant 2-mA current via one 5 × 5-cm anodal electrode positioned on the scalp over the C3 or C4 positions (motor cortex) of the lesioned hemisphere and one cathodal electrode positioned at the ipsilateral shoulder in two 20-min sessions per day for 5 days. The period of follow-up will be 4 weeks.

**Study outcome(s):**

The primary outcome measure will be a change in fatigue severity, as measured using the modified fatigue impact scale (MFIS). The participants’ scores on the MFIS (total score and physical, cognitive and psychosocial subscores) will be collected before treatment (T0), after 10 treatment sessions, i.e., 1 day after the fifth treatment day (T1), and 1 week (T2), 2 weeks (T3) and 4 weeks (T4) thereafter. Both per-protocol analysis and intention-to-treat analysis will be performed.

**Discussion:**

This proposed project will provide proof-of-concept, i.e., demonstrate the benefits of tDCS for the treatment of PSF. The beneficiaries are the subjects participated in the study. This will stimulate further research to optimize tDCS parameters for the treatment of PSF.

**Clinical trial registration:**

www.Chictr.org.cn, identifier: ChiCTR2100052515.

## Introduction

Fatigue is defined as the “subjective lack of physical or mental energy to carry out usual and desired activities as perceived by the patient” (1). Patients with fatigue may experience a devastating sense of tiredness, exhaustion or lack of energy during or after mental or motor activity. Fatigue is a common symptom of neurological disorders (2).

Post-stroke fatigue (PSF) is a common and chronic problem (3), with a frequency that varies from 23 to 85% (3–7). For many stroke patients with good recovery, PSF is the sole major disability (8). It was found that up to 40% of stroke patients reported PSF as the most troublesome consequence of stroke (3). PSF can hinder rehabilitation (5) and predicts reduced functional independence, risk of institutionalization, impairment of cognition, poor quality of life and increased mortality (9). Female sex, older age and previous stroke are clinical correlates of PSF (9). Other causes of PSF include sleep apnea (10) and depression (3, 11).

### Pathological mechanism of PSF

PSF is a complex problem with several contributing factors, many of which are not well understood. One theory holds that stroke may trigger biochemical imbalances, such as inflammatory responses, that generate fatigue in the early stages following stroke. Subsequently, neurophysiological and behavioral perturbations, such as beliefs about estimated action cost, may result in chronic PSF (11).

### Potential treatments that have been explored for alleviation of PSF

Potential pharmacological treatments for PSF, such as antidepressants, stimulants, vitamin D and wakefulness-promoting agents (e.g., modafinil), have failed to demonstrate significant benefits (12). Similarly, the efficacy of non-pharmacological treatments, such as low-intensity training, cognitive therapy, fatigue education, a mindfulness-based stress-reduction program, treatment of associated depression, Chinese herbs, correction of risk factors and adaptation of activities, remains unproven (13).

### Nature of transcranial direct current stimulation (tDCS)

tDCS can regulate sensory, cognitive, motor and behavioral responses (14), as it involves the application of a small amount of current to the cortex that regulates the activity of neurons and induces protracted after-effects in brain function. During tDCS, an anodal electrode is positioned over the targeted area and a cathodal electrode is positioned on a different part of the scalp or another body part, such as the shoulder. Importantly, tDCS does not cause severe adverse effects and is considered to be safe (15) and is a cost-effective and portable technique for neuromodulation (16).

Diverse effects on brain excitability can be achieved via tDCS, depending on the intensity, polarity and duration of the applied electric current. tDCS is a technique that in the short term is capable of modulating the resting potential of the membrane and in the long term of inducing for example functional plastic changes in the networks such as an increase or decrease in synaptic efficacy (17, 18).

The immediate effects of tDCS are caused by the modulation of resting membrane potential, whereas its long-lasting after-effects are due to the induction of enduring depression and potentiation (19). At the single-neuron level, tDCS generates glutamatergic plasticity that modulates the function of various ion channels and of neurotransmitters such as brain-derived neurotrophic factor and *N*-methyl-d-aspartate glutamate (19). In summary, tDCS can modulate neuronal excitability and induce prolonged functional changes in the brain (20). Stimulation of different levels of the neuromotor control system affects neuronal circuits in stroke patients, which causes neuroplastic changes. This reorganizes the damaged brain and produces improvements in function (19).

### Effects of tDCS in stroke patients

There is growing interest in tDCS as a treatment to facilitate stroke recovery, and small-scale clinical trials have suggested that tDCS can be effective in the treatment of various motor and non-motor dysfunctions in stroke patients. For example, tDCS has been found to improve the capacity for activities of daily living (21), swallowing function (22), gait training (23), motor learning (24), visuospatial neglect (25), central pain (26), verbal learning (27) and depression (28) in stroke patients. tDCS may be more effective in patients with stroke with mild-to-moderate impairments than in those with major impairments (29).

### Effects of tDCS in patients with PSF

An open-label trial of two sessions of tDCS per week for 5–6 weeks in 10 stroke patients did not result in any changes in PSF (30). Another recent clinical trial on 30 patients with PSF showed that compared with sham stimulation, one session of anodal tDCS over the bilateral primary motor cortex reduced symptoms of PSF for up to 7 days following treatment (31). One randomized control trial revealed no add-on effect of six sessions of tDCS on fatigue in 74 chronic stroke patients (32). Another randomized control trial of 6 sessions of tDCS per week for 4 weeks in 60 stroke patients reduced fatigue (33). Finally, there is an ongoing trial of six sessions of tDCS in 100 patients with PSF (34).

### tDCS for treatment of fatigue related to neurological disease

tDCS has been explored as a potential treatment for fatigue associated with other neurological diseases, namely, multiple sclerosis (MS), Parkinson’s disease and post-polio syndrome. For example, in a series of studies involving more than 50 patients with MS who received 5 sessions of tDCS applied over the motor cortex (35) or the somatosensory cortex (36, 37), results showed tDCS treatment led to a reduction of fatigue symptoms (35–37) and the improvement might persist up to 3 weeks (35). In a fourth study, 27 patients with MS were given 20 sessions of tDCS over the left dorsolateral prefrontal cortex, which which led to a decrease in their fatigue symptoms (37). In a fifth study, 17 patients with MS were given 10 sessions of random noise stimulation over the primary motor cortex, which led to a decrease in their fatigue symptoms (38). In two studies on patients with post-polio fatigue, 10 (39) and 15 (40) daily sessions of tDCS were found to decrease patients’ fatigue symptoms. In a clinical trial involving 23 patients with Parkinson’s disease, eight daily sessions of tDCS were found to decrease patients’ fatigue symptoms (41). Crucially, mild side effects (headache and tingling sensations) but no serious side-effects have been reported in the above-mentioned studies.

It has been suggested that three neurophysiological mechanisms underlie central fatigue: slowed conduction in central motor pathways, resulting in decreased recruitment of spinal motoneurons; blockage of conduction at Ranvier’s nodes; and impairment in the prefrontal cortex, which is responsible for motor planning (35). tDCS may alleviate PSF through several mechanisms. First, tDCS may restore activation in prefrontal and motor areas (35). Second, tDCS may improve connectivity between motor and frontal areas and the thalamus (35). Third, tDCS may increase positive mood and alleviate depressive symptoms (41).

In a recent review the effect of tDCS on fatigue in neurological disorders, 42 studies were identified. These studies included a total of 994 participants. Amongst these 42 studies, five of them, involving 290 subjects, examined the effect of tDCS in PSF. In 36 out of 42 (85.7%) of studies reported an improvement in fatigue scores in the tDCS group. Side effects of tDCS are usually mild (42). Hence tDCS proves to have a promising simple, low-cost, portable, non-invasive and risk-free procedure for reducing fatigue symptoms in alleviating PSF. The objective of this proposed project will be to examine the effect of tDCS on PSF. We hypothesize that active tDCS is more effective than sham tDCS.

## Methods

### Design

**Figure 1:**
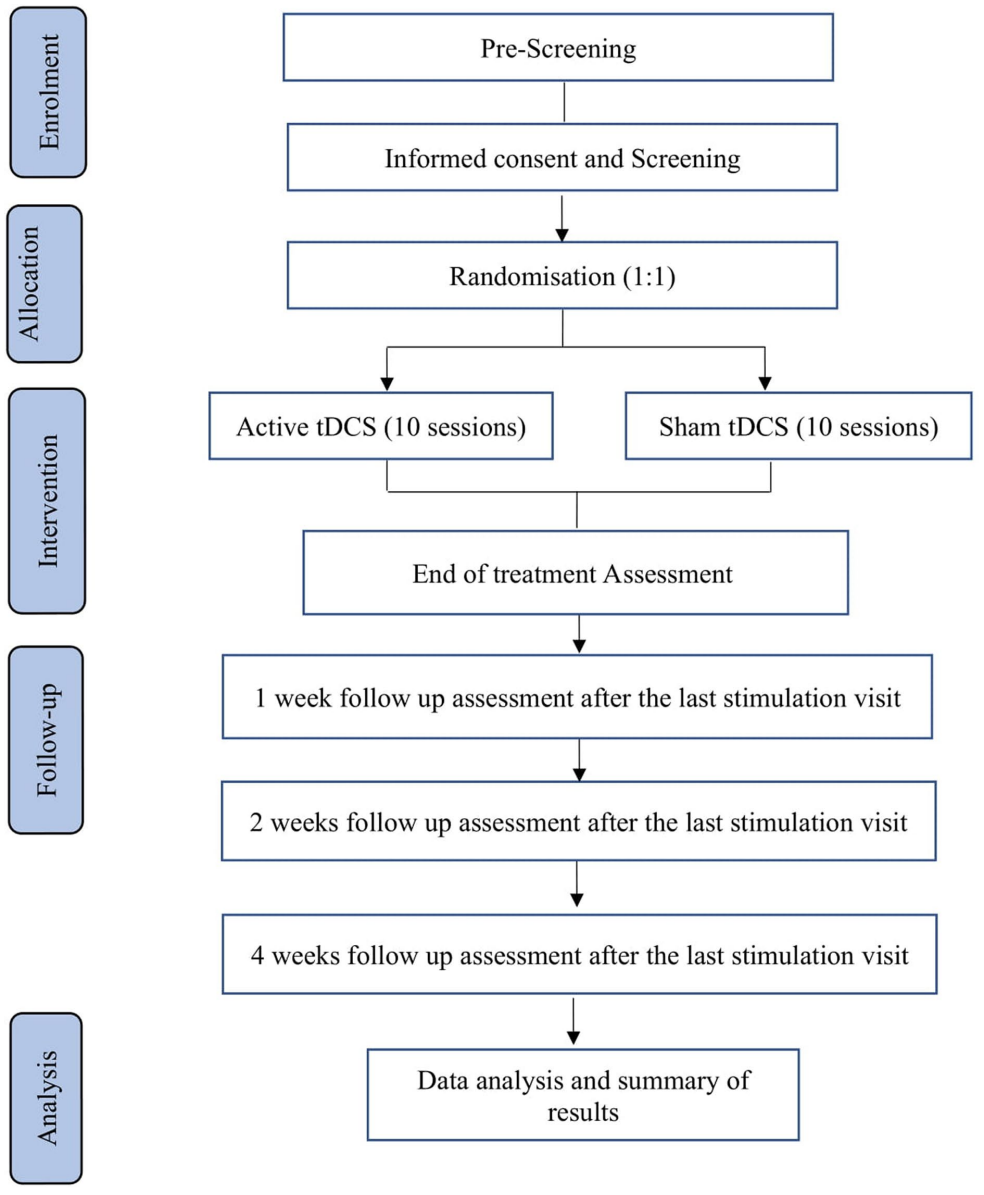
Flow chart of the study.

A double-blind randomized control trial of stroke survivors (Figure 1).

### Patient population

Patients with chronic stroke will be recruited from the Neurology Unit of the Prince of Wales Hospital and the rehabilitation wards of Shatin Hospital (both in Hong Kong) and from the Hong Kong community through newspaper advertisements and word of mouth. A research assistant will visit the above facilities weekly to identify all eligible patients and obtain their written consent.

### Inclusion and exclusion criteria

The *inclusion criteria* will be (1) either sex; (2) aged 18–80; (3) any level of literacy or education; (4) a history of stroke confirmed by an brain imaging examination such as a CT scan or Magnetic Resonance Imaging; (5) able to speak ethnic Chinese and Cantonese; (6) willing and able to give informed consent; and (6) presence of PSF (a Fatigue Severity Scale [FSS] score of 4.0 or more) (43–45). The cut-off score of 4.0 was chosen since it the most commonly used cut-off score. In a recent systematic review of 31 studies using FSS to examine the prevalence of PSF, all of them reported a cut-off point of 4 or more (46).

The *exclusion criteria* will be (1) acute and subacute stroke (≤6 months after onset); (2) a history of depression (self-report or a Beck Depression Inventory (BDI) – II) (47) score ≥ 14; (3) a presence of schizophrenia, bipolar affective disorder, and/or substance/alcohol dependence/abuse; (4) a history of any neurological disorder (except stroke); (5) a history of sleep apnea, narcolepsy, hypothyroidism, heart failure, chronic obstructive pulmonary disease or cancer; (6) a history of unexplained spells of fainting; (7) current pregnancy; (8) a history of neurosurgery; (9) presence of dementia, defined as a Hong Kong version of the Montreal Cognitive Assessment (MoCA) score of less than 19 (48); (10) presence of aphasia, defined as a score > 0 in the best language instructions item of the National Institute of Health Stroke Scale (NIHSS) (49); (11) presence of major depression, as confirmed by a research assistant using the Structured Clinical Interview for Diagnostic and Statistical Manual (DSM) – Fourth Edition (SCID-DSMIV) (50) and according to the DSM – Fifth Edition Diagnostic Criteria (51); (12) taking hypnotics or other medications that can cause fatigue; (13) taking antiepileptic or other medications that can weaken the effect of tDCS; (14) current or past use of tDCS; (15) contraindications to tDCS, e.g., skin damage at the proposed site of stimulation or presence of a pacemaker, a metal implant in the head or a medical device in the brain; and (16) participation in rehabilitative therapy or physical exercise program during the trial. The above criteria will be assessed by examination of patients’ medical record.

### Measurement overview

The data collection schedule is detailed in Table 1. The number of patients excluded and the reasons for their exclusion will be recorded. Data on sex, age, level of education, risk factors of vascular diseases (e.g., smoking, hyperlipidemia, diabetes mellitus and hypertension), date of stroke onset and length of stay in rehabilitation facilities will be collected from all of the participants. Neurological impairment (i.e., the NIHSS total score data) at admission will be extracted from the stroke registry, which will be maintained by a full-time, well-trained research nurse who will also be responsible for administration of the NIHSS.

**Table 1:** Data collection schedule.

Study period	Screening	Treatment	EOT	FUV
**Visit**	**1**	**2–6**	**7**	**8–10**
**Weeks after Randomization**	**-2**	**1**	**1**	**2,3,5**
Review of inclusion / exclusion criteria	X			
Informed consent	X			
Demographics, vascular risk factors, stroke characteristics, previous treatment of PSF	X			
Montreal Cognitive Assessment	X			
MFIS, FFS, PSAE, BI, GDS, HADSA, SSQoL	X		X	X
tDCS		X		
Randomization	X			
tDCS adverse effects questionnaire		X	X	
Experience, preferences, concerns and belief on tDCS questionnaire			X	

Screen denotes Screening visit.

EOT denotes End of treatment.

FUV denotes Follow up visits.

### Baseline measures

A research assistant will assess the participants’ PSF by administering the validated Chinese version of FSS and the MFIS (52–55, 56). The FSS is the most frequently used tool to measure PSF (57–59) and consists of nine Likert items scored on a 7-point scale (e.g., “I am easily fatigued,” “My motivation is lower when I am fatigued than when I am not” and “Exercise brings on my fatigue”). A higher score on an item indicates the presence of greater fatigue, and the scores for all items are averaged to give a total fatigue score. In an assessment of the FSS in patients with neurological diseases, its Cronbach’s α was 0.90–0.94, its intraclass correlation coefficient was 0.73–0.93, and its correlation coefficients with other fatigue scales were 0.62–0.84, indicating that the FSS has good internal consistency, reliability and convergent validity in such patients (58). In addition, information on previous treatment of PSF will be collected using a questionnaire.

The MFIS is administered via a 5–10-min interview that assesses how fatigue affects people’s daily lives, in terms of their psychosocial, cognitive and physical functioning. The MFIS contains 21 items that are scored from 0 to 4, which represent different frequencies, i.e., “never,” “seldom,” “sometimes,” “often” and “always,” respectively. Subscores are obtained for cognitive functioning (0–36), physical functioning (0–40) and psychosocial functioning (0–8) and are summed to give a total score (0–84). A higher score indicates severer fatigue. The MFIS has been used to assess PSF (60) and measure outcomes in clincial trials of interventions for fatigue (61), including tDCS (37, 39, 62, 63).

A research assistant will use the Physical Activity Scale for the Elderly (PASE) (64), the Barthel Index (BI) (65), the MoCA, the anxiety subscale of the Chinese version of the Hospital Anxiety Depression Scale (HADSA), the Chinese version of the Geriatric Depression Scale (GDS), the Pittsburgh Sleep Quality Index (PSQI) (66) and the Chinese version of the Stroke-Specific Quality of Life (SSQoL) scale to, respectively, assess the participants’ level of physical activity, level of disability, global cognitive function, anxiety symptoms, depressive symptoms, sleep disturbances and health-related quality of life. The anxiety subscale of the HADSA consists of seven items, each of which is scored on a 4-point Likert scale, with higher scores representing severer anxiety. The Chinese version of the HADSA has been previously validated (67). The Chinese version of GDS contains 15 items, has a maximum possible score of 15, and has been previously validated (68). The PSQI measures seven components across 19 items, each of which is rated on a scale from 0 to 3, and the total possible score ranges from 0 to 21.

The Chinese version of the SSQoL scale (69, 70) is designed to measure quality of life in stroke survivors. It is a self-report questionnaire that contains 49 items covering 12 domains: family roles (three items), energy (three items), mobility (six items), language (five items), mood (five items), personality (three items), social roles (five items), self-care (five items), upper extremity function (five items), thinking (three items), work/productivity (three items) and vision (three items). Individual items are scored on a 5-point Likert scale representing answers from “completely true” to “not true at all,” with higher scores implying a better quality of life. Thus, the Chinese version of the SSQoL scale generates both domain and total scores. It has been previously shown to have excellent internal consistency, intertest reliability and test–retest reliability (69).

### Randomization and blinding procedure

A statistician will apply a block randomization procedure to afford a concealed randomization list. As mentioned, there with be 78 participants in each group (the sham tDCS group and the active tDCS group). Upon recruitment of each participant, the technician responsible for the tDCS delivery will receive information on the participant’s assigned treatment.

### Intervention

**Figure 2:**
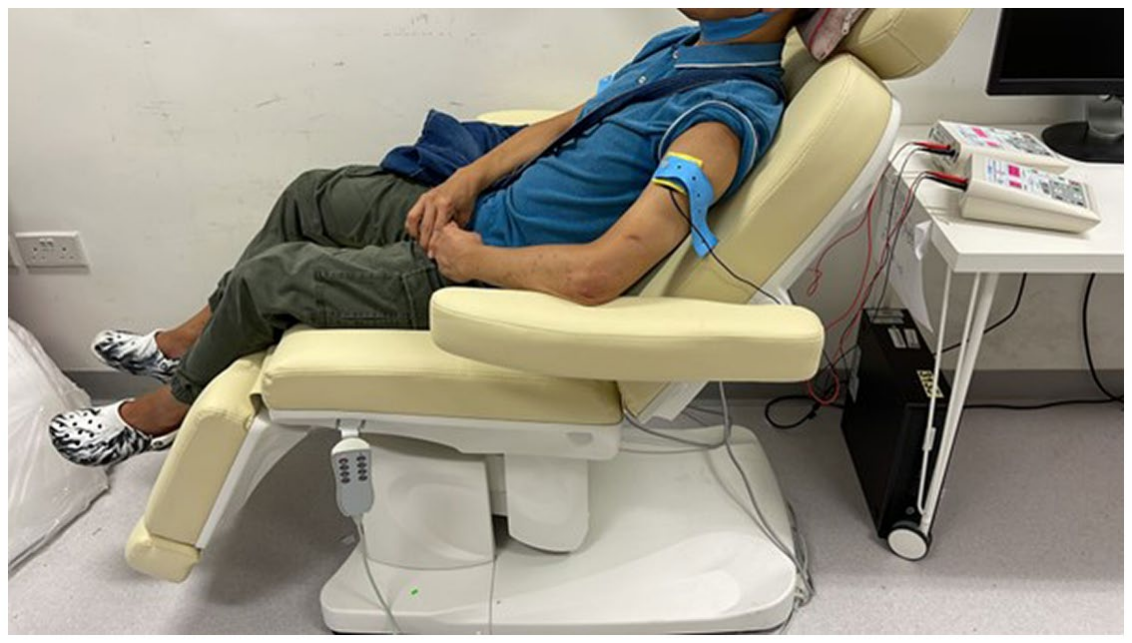
Demonstration of the setup on the arm.

**Figure 3:**
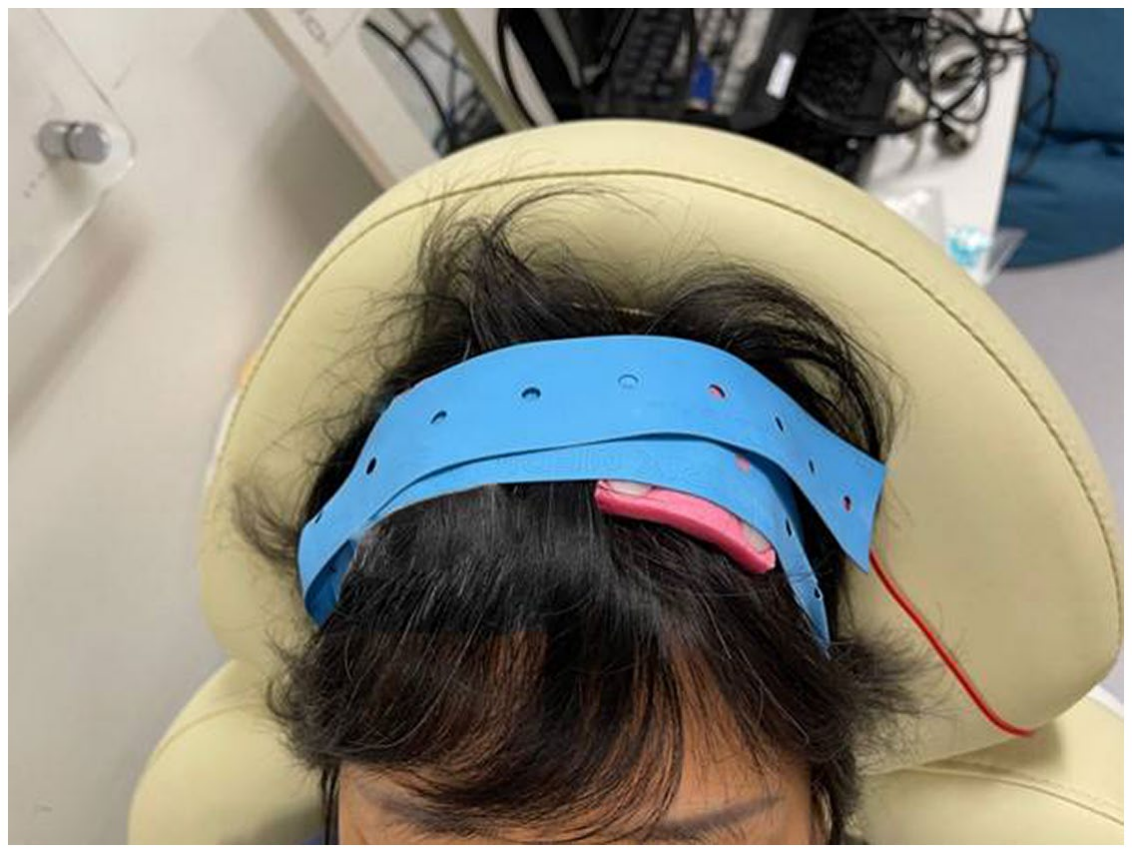
Demonstration of the setup on the scalp (C3).

A trained research assistant will administer tDCS at Shatin Hospital. The participants will recline on a comfortable chair with their eyes closed during tDCS, which will be delivered by one battery-driven constant-current stimulator (Soterix Medical 1 × 1 Clinical Trials Device) through one 5 × 5-cm conductive-rubber anodal electrode. The electrodes will be inserted into an EASYpad (Soterix Medical), which contains sponge material and will be moistened by application of approximately 10–15 mL of saline. The EASYpad will examined every minute to check whether additional saline needs to be added to prevent it from drying out. Each participant will be allocated a set of EASYpads that will be used for the entire treatment course to maintain good hygiene. Elastic straps will be used to fasten the EASYpad to the scalp. The anodal electrode will be positioned on the scalp over the C3 or C4 positions (motor cortex) of the lesioned hemisphere, based on the international electroencephalogram 10/20 system, and a cathodal electrode will be positioned on the ipsilateral shoulder (34) (Figures 2, 3). A constant current of 2 mA (with a current density of 0.08 mA/cm^2^) will be applied to the anode by one of the constant-current stimulators in two 20-min sessions, separated by a 10-min break (34), every day for 5 consecutive days. Sham tDCS will consist of 30 s of constant current at the beginning and end of each 20-min session, such that all of the participants will experience the initial itching sensation at the beginning of stimulation. The participants will be asked about skin sensations they experienced during and at the end of the stimulation to determine if they are able to distinguish between sham and real stimulation. The above stimulation site and parameters were chosen based on the following rationale. First, association observed between increased fatigue scores and decreased functional connectivity of supplementary and primary motor areas (42). In addition, stimulation of motor cortex has been shown to improve fatigue in stroke patients and healthy individuals (31, 71). Second, the duration of stimulation per session was 20 min in all five published studies of tDCS on PSF (42). Third, consecutive sessions of tDCS has been employed were employed in previous studies of tDCS on PSF (32, 34, 71).

### Safety measures

The Adverse Event Checklist (AEC) will be administered at the end of each stimulation session. The AEC covers adverse events (AEs; symptoms) in various systems, namely, the circulatory, digestive, cardiovascular, musculoskeletal, metabolic, urogenital and nervous systems, and the whole body (72).

### Withdrawal criteria

The participants should be able to complete the 10 sessions of tDCS treatment within 1 week. If they miss a scheduled session, they can receive another session when convenient. If the participants cannot complete the 10 sessions, they will be regarded as dropouts. In addition, participants will be removed from the study if they (i) withdraw their consent for tDCS treatment and/or (ii) have a severe AE that may lead to significant distressful consequences (seizure, skin burn or blister).

We will continue to follow withdrawn participants and perform outcome measures as far as possible.

### Primary outcome measure

The MFIS will be administered before treatment (T0), after 10 treatment sessions (T1, 1 day after the fifth treatment day), and 1 week (T2), 2 weeks (T3) and 4 weeks (T4) thereafter (36), at the same time of day. The time window of administration will be adjusted to assess changes between time points (4 weeks for T0, 1 day for T1, 1 week for T2 and T3, and 2 weeks for T4). The participants and the research assistant who administers the MFIS will be blinded to the delivered treatment.

### Secondary outcome measures

The FSS, the Chinese version of the GDS, the Chinese version of the HADSA, the PSQI, the BI and the PASE will be administered at T0, T1, T2, T3, and T4 (36), at the same time of day. The Chinese version of the SSQoL will be administered at T0 and T4. The time window of administration will be adjusted to assess changes between time points, as stated above. Data on the participants’ experience of, preferences for, and concerns and beliefs about tDCS will be collected at T4 via a brief questionnaire (33).

### Data monitoring body

An independent Data Safety Monitoring Board will be formed (DSMB) and will consist of a stroke neurologist, a psychiatrist, a statistician and a tDCS clinical trial specialist. The DSMB will meet regularly and examine AEs, perform an interim analysis and make recommendations on the safety of the study and the need to stop or extend the study.

### Sample size estimates

Statistical power was computed using Power Analysis & Sample Size 2005. We assumed that the standard deviation of the MFIS score at baseline will be 3.9 (60), that the sham stimulation will not cause any change in fatigue and that a decrease of at least 3.0 MFIS points will be clinically relevant (36). Hence, the effect size of tDCS will be (3.0/3.9 = 0.77). Given that such a large effect size is unusual in clinical contexts, we will instead use a more realistic effect size, i.e., 0.50. One pairwise comparison will be performed in the analysis of variance. A sample size of 63 per group (a total of 126 participants) will give rise to 80% power in identifying the main effect of tDCS (73). Thus, assuming a dropout rate of 20%, 156 (126/0.8) participants will be recruited.

### Statistical analysis

We will perform a per-protocol analysis and an intention-to-treat analysis. Only participants who complete treatment will be included in the per-protocol analysis, whereas all of the participants who receive at least one tDCS session will be included in the intention-to treat-analysis. A last-observation-carried-forward approach will be adopted for the analysis of outcome data. The mean score of the FSS will be calculated. The sum of individual item scores will be computed for the MFIS and the total score and physical, cognitive and psychosocial subscores will be derived. Demographic characteristics will comprise age, sex, and scores on the Chinese version of the GDS, the Chinese version of the HADSA, the PASE, the BI, the PSQI, and the NIHSS, and the severity of PSF will be measured using the FSS and the MFIS. Chi-square tests (for categorical variables such as sex) and t-tests (for continuous variables such as age) will be used to compare baseline performance. The effects of tDCS upon completion of treatment (T1) will be studied by entering the participants’ scores on the MFIS (total score and subscores) into a mixed-model analysis of variance with baseline MFIS scores as covariates, and the tDCS intervention (T0, T1) and stimulation (active, sham) as between-participant factors. The trend of tDCS effects will be studied by entering the participants’ scores on the MFIS (total score and subscores) into a linear mixed model with the tDCS intervention (T0, T1, T2, T3 and T4) and stimulation (active, sham) as within-participant factors. Pairwise comparisons between baseline and individual post-measurement-day significant results will conducted using Bonferroni t-tests for related samples. Withdrawals will be analyzed by logistic regression. The level of significance will be 0.05.

The safety analysis will be descriptive and exploratory. Key safety measures will be based on the tDCS AEs questionnaire and include the overall incidence and intensity of AEs; the number of AEs leading to treatment discontinuation; skin reactions; and other AEs.

### Organization and funding

The proposed project will be performed in the acute stroke unit of the Prince of Wales Hospital, Shatin, Hong Kong, China, where research coordination and analyzes will also be conducted. The proposed project has received support from the General Research Fund of the Research Grants Council of Hong Kong, China.

#### Access to data

Only the Principal Investigator (WKT) will have access to the final dataset.

#### Discussion and summary

We will endeavor to obtain a homologous sample by obtaining a set of participants with a narrow range of ages, ethnicities, handedness and duration of PSF. Potential participants with other causes of fatigue, such as depression, psychiatric disorders, alcohol/substance abuse and neurological disorders, will be excluded. We will use two tDCS devices to achieve bilateral stimulation of the motor cortex with a relatively high current and long duration of stimulation. The MFIS will be used to measure the primary outcome (a change in PSF severity), as it is commonly used in clinical trials of interventions for fatigue. A variety of secondary outcomes will be assessed, such as mood, quality of life and functioning. A conservative estimate of effect size has been used to calculate an adequate sample size. On the other hand, exclusion of patients with aphasia will limit the generalizability of the study’s results to this subgroup of patients. This proposed project will provide proof-of-concept, i.e., will demonstrate the benefits of tDCS in the treatment of PSF, thereby stimulating further research to determine the optimal tDCS parameters to alleviate PSF.

## Ethics statement

The studies involving humans were approved by the Joint CUHK-NTEC CREC (CREC: ref 2020.550). (The Chinese University of Hong Kong (CUHK) and New Territories East Cluster (NTEC) Clinical Research Ethics Committee). The studies were conducted in accordance with the local legislation and institutional requirements. The participants provided their written informed consent to participate in this study.

## Author contributions

WT: Writing – original draft. HL: Writing - review & editing, Conceptualization and methodology. TL: Writing – review & editing. JK: Writing – review & editing. KF: Writing – review & editing.

## References

[1] Rammohan KW Rosenberg JH Lynn DJ Blumenfeld AM Pollak CP Nagaraja HN. Efficacy and safety of modafinil (Provigil) for the treatment of fatigue in multiple sclerosis: a two Centre phase 2 study. J Neurol Neurosurg Psychiatry. (2002) 72:179–83. doi: 10.1136/jnnp.72.2.179, PMID: 11796766 PMC1737733

[2] Penner I-K Paul F. Fatigue as a symptom or comorbidity of neurological diseases. Nat Rev Neurol. (2017) 13:662–75. doi: 10.1038/nrneurol.2017.117 29027539

[3] Paciaroni M Acciarresi M. Poststroke Fatigue. Stroke. (2019) 50:1927–33. doi: 10.1161/STROKEAHA.119.023552 31195940

[4] Van Zandvoort MJE Kappelle LJ Algra A De Haan EHF. Decreased capacity for mental effort after single supratentorial lacunar infarct may affect performance in everyday life. J Neurol Neurosur Ps. (1998) 65:697–702. doi: 10.1136/jnnp.65.5.697, PMID: 9810940 PMC2170367

[5] Ingles JL Eskes GA Phillips SJ. Fatigue after stroke. Arch Phys Med Rehabil. (1999) 80:173–8. doi: 10.1016/S0003-9993(99)90116-8 10025492

[6] Staub F Bogousslavsky L. Fatigue after stroke: a major but neglected issue. Cerebrovasc Dis. (2001) 12:75–81. doi: 10.1159/000047685 11490100

[7] Tang WK Chen YK Mok V Chu WC Ungvari GS Ahuja AT . Acute basal ganglia infarcts in poststroke fatigue: an MRI study. J Neurol. (2010) 257:178–82. doi: 10.1007/s00415-009-5284-2, PMID: 19688358

[8] Chaudhuri A Behan PO. Fatigue in neurological disorders. Lancet. (2004) 363:978–88. doi: 10.1016/S0140-6736(04)15794-2 15043967

[9] Glader EL Stegmayr B Asplund K. Poststroke fatigue: a 2-year follow-up study of stroke patients in Sweden. Stroke. (2002) 33:1327–33. doi: 10.1161/01.STR.0000014248.28711.D6 11988611

[10] Sandberg O Franklin KA Bucht G Gustafson Y. Sleep apnea, delirium, depressed mood, cognition, and ADL ability after stroke. J Am Geriatr Soc. (2001) 49:391–7. doi: 10.1046/j.1532-5415.2001.49081.x, PMID: 11347781

[11] De Doncker W Dantzer R Ormstad H Kuppuswamy A. Mechanisms of poststroke fatigue. J Neurol Neurosurg Psychiatry. (2018) 89:287–93. doi: 10.1136/jnnp-2017-316007 28939684

[12] Kennedy C Kidd L. Interventions for post-stroke fatigue: a Cochrane review summary. Int J Nurs Stud. (2018) 85:136–7. doi: 10.1016/j.ijnurstu.2017.11.006 29525448

[13] Wu SM Kutlubaev MA Chun HYY Cowey E Pollock A Macleod MR . Interventions for post-stroke fatigue. Cochrane Db Syst Rev. (2015) 2015:CD007030. doi: 10.1002/14651858.CD007030.pub3 PMC7387276 26133313

[14] Vallar G Bolognini N. Behavioural facilitation following brain stimulation: implications for neurorehabilitation. Neuropsychol Rehabil. (2011) 21:618–49. doi: 10.1080/09602011.2011.574050, PMID: 21604229

[15] Poreisz C Boros K Antal A Paulus W. Safety aspects of transcranial direct current stimulation concerning healthy subjects and patients. Brain Res Bull. (2007) 72:208–14. doi: 10.1016/j.brainresbull.2007.01.004, PMID: 17452283

[16] Lu H Chan SSM Chan WC Lin C Cheng CPW Wa LC . Randomized controlled trial of TDCS on cognition in 201 seniors with mild neurocognitive disorder. Ann Clin Transl Neurol. (2019) 6:1938–48. doi: 10.1002/acn3.50823, PMID: 31529691 PMC6801176

[17] Furubayashi T Terao Y Arai N Okabe S Mochizuki H Hanajima R . Short and long duration transcranial direct current stimulation (tDCS) over the human hand motor area. Exp Brain Res. (2007) 185:279–86. doi: 10.1007/s00221-007-1149-z, PMID: 17940759

[18] Hodkinson DJ Jackson SR Jung J. Task-dependent plasticity in distributed neural circuits after transcranial direct current stimulation of the human motor cortex: a proof-of-concept study. Front. Pain Res. (2022) 3:1005634. doi: 10.3389/fpain.2022.1005634, PMID: 36506269 PMC9732378

[19] Bao SC Khan A Song R Kai-Yu TR. Rewiring the lesioned brain: electrical stimulation for post-stroke motor restoration. J Stroke. (2020) 22:47–63. doi: 10.5853/jos.2019.03027, PMID: 32027791 PMC7005350

[20] Schlaug G Renga V Nair D. Transcranial direct current stimulation in stroke recovery. Arch Neurol. (2008) 65:1571–6. doi: 10.1001/archneur.65.12.1571, PMID: 19064743 PMC2779259

[21] Elsner B Kugler J Pohl M Mehrholz J. Transcranial direct current stimulation (tdcs) for improving activities of daily living, and physical and cognitive functioning, in people after stroke. Cochrane Database Syst Rev. (2020) 11:CD009645. doi: 10.1002/14651858.CD009645.pub4 33175411 PMC8095012

[22] Shigematsu T Fujishima I Ohno K. Transcranial direct current stimulation improves swallowing function in stroke patients. Neurorehabil Neural Repair. (2013) 27:363–9. doi: 10.1177/1545968312474116 23392916

[23] Picelli A Chemello E Castellazzi P Roncari L Waldner A Saltuari L . Combined effects of transcranial direct current stimulation (tDCS) and transcutaneous spinal direct current stimulation (tsDCS) on robot-assisted gait training in patients with chronic stroke: a pilot, double blind, randomized controlled trial. Restor Neurol Neurosci. (2015) 33:357–68. doi: 10.3233/RNN-140474, PMID: 26410579

[24] Kang N Summers JJ Cauraugh JH. Transcranial direct current stimulation facilitates motor learning post-stroke: a systematic review and meta-analysis. J Neurol Neurosurg Psychiatry. (2016) 87:345–55. doi: 10.1136/jnnp-2015-311242, PMID: 26319437

[25] Bang DH Bong SY. Effect of combination of transcranial direct current stimulation and feedback training on visuospatial neglect in patients with subacute stroke: a pilot randomized controlled trial. J Phys Ther Sci. (2015) 27:2759–61. doi: 10.1589/jpts.27.2759, PMID: 26504287 PMC4616088

[26] Bae SH Kim GD Kim KY. Analgesic effect of transcranial direct current stimulation on central post-stroke pain. Tohoku J Exp Med. (2014) 234:189–95. doi: 10.1620/tjem.234.189, PMID: 25341455

[27] Yun GJ Chun MH Kim BR. The effects of transcranial direct-current stimulation on cognition in stroke patients. J Stroke. (2015) 17:354–8. doi: 10.5853/jos.2015.17.3.354, PMID: 26438001 PMC4635724

[28] Li Y Li H-P Wu M-X Wang Q-Y Zeng X. Effects of transcranial direct current stimulation for post-stroke depression: a systematic review and meta-analysis. Clin Neurophysiol. (2022) 142:1–10. doi: 10.1016/j.clinph.2022.07.369, PMID: 35914485

[29] Baltar A Piscitelli D Marques D Shirahige L Monte-Silva K. Baseline motor impairment predicts transcranial direct current stimulation combined with physical therapy-induced improvement in individuals with chronic stroke. Neural Plast. (2020) 2020:1–8. doi: 10.1155/2020/8859394 PMC7710411 33299400

[30] Cleland BT Galick M Huckstep A Lenhart L Madhavan S. Feasibility and safety of transcranial direct current stimulation in an outpatient rehabilitation setting after stroke. Brain Sci. (2020) 10:719. doi: 10.3390/brainsci10100719, PMID: 33050340 PMC7599981

[31] De Doncker W Ondobaka S Kuppuswamy A. Effect of transcranial direct current stimulation on post-stroke fatigue. J Neurol. (2021) 268:2831–42. doi: 10.1007/s00415-021-10442-8, PMID: 33598767 PMC8289762

[32] Ulrichsen KM Kolskår KK Richard G Pedersen ML Alnæs D Dørum ES . No add-on effect of tDCS on fatigue and depression in chronic stroke patients: a randomized sham-controlled trial combining tDCS with computerized cognitive training. Brain Behav. (2022) 12:e2643. doi: 10.1002/brb3.2643, PMID: 35666655 PMC9304833

[33] Dong X-L Sun X Sun W-M Yuan Q Yu G-H Shuai L . A randomized controlled trial to explore the efficacy and safety of transcranial direct current stimulation on patients with post-stroke fatigue. Medicine. (2021) 100:e27504. doi: 10.1097/MD.0000000000027504, PMID: 34731132 PMC8519229

[34] Sun X Dong X Yuan Q Yu G Shuai L Ma C . Effects of transcranial direct current stimulation on patients with post-stroke fatigue: a study protocol for a double-blind randomized controlled trial. Trials. (2022) 23:200. doi: 10.1186/s13063-022-06128-9, PMID: 35248120 PMC8898477

[35] Ferrucci R Vergari M Cogiamanian F Bocci T Ciocca M Tomasini E . Transcranial direct current stimulation (tDCS) for fatigue in multiple sclerosis. NeuroRehabilitation. (2014) 34:121–7. doi: 10.3233/NRE-131019 24284464

[36] Tecchio F Cancelli A Cottone C Zito G Pasqualetti P Ghazaryan A . Multiple sclerosis fatigue relief by bilateral somatosensory cortex neuromodulation. J Neurol. (2014) 261:1552–8. doi: 10.1007/s00415-014-7377-9, PMID: 24854634

[37] Charvet LE Dobbs B Shaw MT Bikson M Datta A Krupp LB. Remotely supervised transcranial direct current stimulation for the treatment of fatigue in multiple sclerosis: results from a randomized, sham-controlled trial. Mult Scler. (2018) 24:1760–9. doi: 10.1177/1352458517732842, PMID: 28937310 PMC5975187

[38] Salemi G Vazzoler G Ragonese P Bianchi A Cosentino G Croce G . Application of tRNS to improve multiple sclerosis fatigue: a pilot, single-blind, sham-controlled study. J Neural Transm. (2019) 126:795–9. doi: 10.1007/s00702-019-02006-y, PMID: 31054015

[39] Curatolo M La Bianca G Cosentino G Baschi R Salemi G Talotta R . Motor cortex tRNS improves pain, affective and cognitive impairment in patients with fibromyalgia: preliminary results of a randomised sham-controlled trial. Clin Exp Rheumatol. (2017) 35 Suppl 105(3):100–5. 28681715

[40] Acler M Bocci T Valenti D Turri M Priori A Bertolasi L. Transcranial direct current stimulation (tDCS) for sleep disturbances and fatigue in patients with post-polio syndrome. Restor Neurol Neurosci. (2013) 31:661–8. doi: 10.3233/RNN-130321, PMID: 23863351

[41] Forogh B Rafiei M Arbabi A Motamed MR Madani SP Sajadi S. Repeated sessions of transcranial direct current stimulation evaluation on fatigue and daytime sleepiness in Parkinson’s disease. Neurol Sci. (2016) 38:249–54. doi: 10.1007/s10072-016-2748-x, PMID: 27796604

[42] Jagadish A Shankaranarayana AM Natarajan M Solomon JM. Transcranial direct current stimulation for fatigue in neurological conditions: a systematic scoping review. Physiother Res Int. (2023) 15:e254. doi: 10.1002/pri.2054 37838979

[43] Schwartz JE Jandorf L Krupp LB. The measurement of fatigue: a new instrument. J Psychosom Res. (1993) 37:753–62. doi: 10.1016/0022-3999(93)90104-N 8229906

[44] Chen SXYF . Syndrome differentiation and treatment of post-stroke fatigue. J Pract Tradit Chin Int Med. (2008) 22:17–9.

[45] Tang WK Lau CG Mok V Ungvari GS Wong KS. The impact of pain on health-related quality of life 3 months after stroke. Top Stroke Rehabil. (2015) 22:194–200. doi: 10.1179/1074935714Z.0000000024 25906672

[46] Alghamdi I Ariti C Williams A Wood E Hewitt J. Prevalence of fatigue after stroke: a systematic review and meta-analysis. Eur Stroke J. (2021) 6:319–32. doi: 10.1177/23969873211047681, PMID: 35342803 PMC8948505

[47] Beck AT Steer RA Ball R Ranieri WF. Comparison of Beck depression inventories-IA and -II in psychiatric outpatients. J Pers Assess. (1996) 67:588–97. doi: 10.1207/s15327752jpa6703_13, PMID: 8991972

[48] Yeung PY Wong LL Chan CC Yung CY Leung LJ Tam YY . Montreal cognitive assessment — single Cutoff achieves screening purpose. Neuropsychiatr Dis Treat. (2020) 16:2681–7. doi: 10.2147/NDT.S269243, PMID: 33192067 PMC7656779

[49] Lyden P . Using the National Institutes of Health stroke scale. Stroke. (2017) 48:513–9. doi: 10.1161/STROKEAHA.116.015434 28077454

[50] So E Kam I Leung CM Chung D Liu Z Fong S. The Chinese-bilingual SCID-I/P project: stage 1 — reliability for mood disorders and schizophrenia. Hong Kong J Psychiatry. (2003) 13:7–18.

[51] American Psychiatric Association . (2013). Diagnostic and statistical manual of mental disorders (DSM-5). American Psychiatric Association.

[52] Multiple Sclerosis Council for Clinical Practice Guidelines . Fatigue and multiple sclerosis: Evidence-based management strategies for fatigue in multiple sclerosis. Washington, DC: Paralyzed Veterans of America (1998).

[53] Chen K Fan Y Hu R Yang T Li K. Impact of depression, fatigue and disability on quality of life in Chinese patients with multiple sclerosis. Stress Health. (2013) 29:108–12. doi: 10.1002/smi.2432, PMID: 22566371

[54] Ng SS Liu TW Tsoh J. Translation and initial validation of Chinese (Cantonese) version of modified fatigue impact scale (mfis-C) in people with stroke. BMC Neurol. (2022) 22:300. doi: 10.1186/s12883-022-02832-w, PMID: 35971081 PMC9377082

[55] Wang M-Y Liu I-C Chiu C-H Tsai P-S. Cultural adaptation and validation of the Chinese version of the fatigue severity scale in patients with major depressive disorder and nondepressive people. Qual Life Res. (2015) 25:89–99. doi: 10.1007/s11136-015-1056-x, PMID: 26115873

[56] Stagg CJ Nitsche MA. Physiological basis of transcranial direct current stimulation. Neuroscientist. (2011) 17:37–53. doi: 10.1177/1073858410386614 21343407

[57] Tang WK Lu JY Chen YK Mok VC Ungvari GS Wong KS. Is fatigue associated with short-term health-related quality of life in stroke? Arch Phys Med Rehabil. (2010) 91:1511–5. doi: 10.1016/j.apmr.2010.06.026, PMID: 20875507

[58] Elbers RG Rietberg MB van Wegen EE Verhoef J Kramer SF Terwee CB . Self-report fatigue questionnaires in multiple sclerosis, Parkinson's disease and stroke: a systematic review of measurement properties. Qual Life Res. (2012) 21:925–44. doi: 10.1007/s11136-011-0009-2, PMID: 22012025 PMC3389599

[59] Tang WK Liang HJ Chen YK Chu WNCW Abrigo J Mok VCT . Poststroke fatigue is associated with caudate infarcts. J Neurol Sci. (2013) 324:131–5. doi: 10.1016/j.jns.2012.10.022, PMID: 23142065

[60] Hubacher M Calabrese P Bassetti C Carota A Stocklin M Penner IK. Assessment of post-stroke fatigue: the fatigue scale for motor and cognitive functions. Eur Neurol. (2012) 67:377–84. doi: 10.1159/000336736, PMID: 22614741

[61] De Giglio L De Luca F Prosperini L Borriello G Bianchi V Pantano P . A low-cost cognitive rehabilitation with a commercial video game improves sustained attention and executive functions in multiple sclerosis: a pilot study. Neurorehabil Neural Repair. (2015) 29:453–61. doi: 10.1177/1545968314554623, PMID: 25398725

[62] Saiote C Goldschmidt T Timaus C Steenwijk MD Opitz A Antal A . Impact of transcranial direct current stimulation on fatigue in multiple sclerosis. Restor Neurol Neurosci. (2014) 32:423–36. doi: 10.3233/RNN-130372 24531295

[63] Cancelli A Cottone C Giordani A Migliore S Lupoi D Porcaro C . Personalized, bilateral whole-body somatosensory cortex stimulation to relieve fatigue in multiple sclerosis. Mult Scler. (2018) 24:1366–74. doi: 10.1177/1352458517720528, PMID: 28756744

[64] Washburn RA Smith KW Jette AM Janney CA. The physical activity scale for the elderly (PASE): development and evaluation. J Clin Epidemiol. (1993) 46:153–62. doi: 10.1016/0895-4356(93)90053-4, PMID: 8437031

[65] Mahoney FI Barthel DW. Functional evaluation: the Barthel index. Md State Med J. (1965) 14:61–5. 14258950

[66] Tsai PS Wang SY Wang MY Su CT Yang TT Huang CJ . Psychometric evaluation of the Chinese version of the Pittsburgh sleep quality index (CPSQI) in primary insomnia and control subjects. Qual Life Res. (2005) 14:1943–52. doi: 10.1007/s11136-005-4346-x, PMID: 16155782

[67] Lam CL Pan P-C Chan AW Chan S-Y Munro C. Can the hospital anxiety and depression (HAD) scale be used on Chinese elderly in general practice? Fam Pract. (1995) 12:149–54. doi: 10.1093/fampra/12.2.149, PMID: 7589936

[68] Chau J Martin CR Thompson DR Chang AM Woo J. Factor structure of the Chinese version of the geriatric depression scale. Psychol Health Med. (2006) 11:48–59. doi: 10.1080/13548500500093688 17129894

[69] Wang YL Ma JG Li JT. The study on reliability,validity andresponsiveness of the Chinese version of stroke-specific Qualityof life. Chin J Geriatr Heart Brain Vessel D. (2003) 5:391–4.

[70] Hsueh IP Jeng JS Lee Y Sheu CF Hsieh CL. Construct validity of the stroke-specific quality of life questionnaire in ischemic stroke patients. Arch Phys Med Rehabil. (2011) 92:1113–8. doi: 10.1016/j.apmr.2011.02.008, PMID: 21704791

[71] Gandiga PC Hummel FC Cohen LG. Transcranial DC stimulation (tDCS): a tool for double-blind sham-controlled clinical studies in brain stimulation. Clin Neurophysiol. (2006) 117:845–50. doi: 10.1016/j.clinph.2005.12.003, PMID: 16427357

[72] Lu H Lam LCW. Cathodal skin lesions induced by transcranial direct current stimulation (tDCS). Neuromodulation. (2019) 22:989–91. doi: 10.1111/ner.12892, PMID: 30506838

[73] Edwards L . Applied analysis of variance in behavioral science. New York, NY: Marcel Dekker, Inc (1993).

